# The Prevalence and Risk Factors of Postpartum Depression Among Saudi Arabian Women—A Systematic Review and Meta-Analysis

**DOI:** 10.3390/healthcare13162040

**Published:** 2025-08-18

**Authors:** Mohamed Zarroug, Mohammed F. Altaf, Safwaan Shaikh, Abdousabour Tidjani, Omnia Bashir, Mohammad I. Ayash, Hana K. Abdalla, Samah H. O. Zarroug

**Affiliations:** 1 School of Public Health, Yale University, New Haven, CT 06520, USA; mohamed.zarroug@aya.yale.edu; 2 College of Medicine, Alfaisal University, Al Takhassousi Road, P.O. Box 50927, Riyadh 11533, Saudi Arabia; maltaf@alfaisal.edu (M.F.A.); smshaikh@alfaisal.edu (S.S.); atidjani@alfaisal.edu (A.T.); obashir@alfaisal.edu (O.B.); mayash@alfaisal.edu (M.I.A.); 3 Department of Microbiology and Immunology, College of Medicine, Alfaisal University, Al Takhassousi Road, P.O. Box 50927, Riyadh 11533, Saudi Arabia; haabdalla@alfaisal.edu; 4 Department of Pharmacology and Therapeutics, College of Medicine, Alfaisal University, Al Takhassousi Road, P.O. Box 50927, Riyadh 11533, Saudi Arabia

**Keywords:** prevalence, risk factors, postpartum depression (PPD), Saudi Arabia

## Abstract

**Background:** Postpartum depression (PPD) is a major public health issue affecting maternal well-being and infant development. This systematic review and meta-analysis aimed to determine the prevalence and risk factors of PPD among Saudi women. **Methods:** A systematic search of PubMed, Web of Science, ProQuest, and EBSCOHost was conducted for studies published up to 31 March 2025. Statistical analysis was performed using R software (v4.4.2) with a random effects model. Study quality was assessed using the Joanna Briggs Institute (JBI) tool. **Results:** A total of 32 studies published between 2014 and 2024, including 10,975 women with a mean age of 30.38 ± 6.22 years, were analyzed. Prevalence of PPD varied based on the cutoff scores of the Edinburgh Postnatal Depression Scale (EPDS): 18% (95% CI: 10–27%) at EPDS ≥ 14, 30% (95% CI: 21–38%) at EPDS ≥ 13, 59% (95% CI: 33–85%) at EPDS ≥ 12, and 45% (95% CI: 28–62%) at EPDS ≥ 10. Across studies, 32 significant risk factors for PPD were identified. The most frequently reported included lack of social support, delivery method, young maternal age, and personal or family history of depression. In the meta-analysis, three factors showed statistically significant associations with postpartum depression: limited family support (*p* < 0.00001), poor spouse support (*p* < 0.00001), and unplanned pregnancy (*p* = 0.04). **Conclusions:** PPD is highly prevalent among Saudi women, with marked regional disparities. These findings highlight the need for tailored mental health strategies and region-specific interventions.

## 1. Introduction

Postpartum depression (PPD) is a prevalent yet frequently underdiagnosed mental illness that occurs in women after childbirth [1,2]. It is defined by persistent sadness, low energy, irritability, a change in sleep or appetite, and inability to bond with the newborn [2,3,4]. PPD is differentiated from the mild, self-limited “baby blues”, occurring in the immediate postpartum period, by both severity and duration, frequently lasting beyond the first few weeks postpartum [5]. Postpartum depression (PPD) is a prevalent yet frequently underdiagnosed mental illness that occurs in women after childbirth, with onset typically within the first few weeks postpartum and symptoms that may persist for up to one year after delivery. Early postpartum depression (EPPD), defined as the onset of depressive symptoms within the first 4–6 weeks following childbirth, holds particular clinical importance, as early symptoms predict persistent postpartum depression, can negatively affect maternal–infant bonding, and may disrupt breastfeeding and caregiving practices. Distinguishing EPPD from later-onset presentations enables the implementation of timely interventions aimed at mitigating adverse psychological and developmental outcomes.

Prevalence of PPD across the world ranges between 10% and 20%, though in low- and middle-income nations it may be higher on account of insufficient access to healthcare, stigma, and social stressors [6]. For the global estimated prevalence of PPD, in a meta-analytic summary by Shorey et al. [7], it was roughly 17.7% with wide variability by geographic location and methodology of the studies. PPD is a public health problem that is severe, in addition to causing significant health and physical issues in the mother, as well as the child’s emotional and cognitive growth. Infants of mothers with PPD who are untreated may develop emotional dysregulation, mental problems, and attachment issues [8]. For example, such children may exhibit increased anxiety, difficulties in social interaction, or delayed language development during early childhood. In addition, PPD that goes untreated may also lead to marital tensions, family maladjustment, and, in extreme cases, maternal suicides [9]. These effects reinforce the importance of early identification and intervention.

In recent years, studies have increasingly emphasized the significant burden of PPD in the Middle East and Gulf nations. Social, religious, and cultural contexts in these countries can potentially be critical in determining women’s mental health in the postpartum window. Underlying mental health literacy, stigma associated with psychological disease, and low utilization of psychiatric care might contribute to PPD underreporting and delay in diagnosis [10,11]. A 2021 meta-analysis by Ahmad et al. [12], which aggregated data across studies conducted between 2006 and 2020 in different Middle Eastern countries, estimated the overall PPD prevalence to be around 27%, significantly higher compared with global estimates. Part of the high rate might be explained by localized determinants of PPD like consanguineous unions, family pressure, lack of social support, and low accessibility of mental health services. While the overview was also critical of discrepancies in methodologies and the variable application of assessment devices, it noted that studies conducted in Gulf Cooperation Council (GCC) countries—such as Saudi Arabia, the United Arab Emirates, and Qatar—consistently reported moderate to high PPD frequencies. However, significant variability across countries outside the GCC was evident, likely reflecting differences in healthcare infrastructure and diagnostic protocols. Therefore, while findings may be indicative of trends within more technologically advanced Gulf nations, caution is warranted in generalizing these outcomes to all Middle Eastern countries, particularly those with differing resource levels or clinical practices. For instance, Abou-Saleh and Ghubash [13] reported a PPD of 18% among Emirati women, whereas newer studies in Qatar and the United Arab Emirates reported between 17.6% and 35% as the prevalence of PPD [14,15].

The Edinburgh Postnatal Depression Scale (EPDS) was the most frequently employed screening instrument among the included studies. The EPDS is a 10-item self-report questionnaire specifically developed to identify depressive symptoms in the postpartum period, with total scores ranging from 0 to 30. The instrument minimizes the influence of somatic symptoms common in the puerperium, thereby improving specificity for depressive symptomatology. Arabic-language versions of the EPDS have been validated in Saudi and other Arabic-speaking populations, demonstrating good internal consistency (Cronbach’s α ≥ 0.80) and construct validity. Cutoff thresholds varied between studies, with scores ≥ 10 frequently used to indicate possible depression and scores ≥ 13 or ≥14 indicating probable major depression.

The Beck Depression Inventory (BDI), a 21-item measure of depressive symptom severity, was also utilized in some studies. Although not specific to the postpartum period, the BDI is a well-validated and widely applied instrument for the assessment of depression in general populations.

In Saudi Arabia, PPD has attracted greater research interest in the last two decades, although findings are highly variable and are frequently reported without generalizability. Prevalence estimates in individual studies varied between as low as 14% and as high as greater than 40%, varying by population recruited, postpartum assessment timing, and screening instrument utilized [16,17]. Such heterogeneity in findings makes estimation of the true burden of PPD in the population at the national level difficult. Saudi Arabia’s specific cultural and societal composition—large family size, role-specific by gender, and changing dynamics surrounding the employment and autonomy of women—may influence both the experience of PPD, as well as how risk factors for it are identified. For example, potential risk factors of poor support in the marriage, unplanned status of the pregnancy, low socioeconomic status, and prior history of psychiatric illness were reported in various local studies, yet there is no agreed-upon synthesis to allow quantification of the strength of association between them [18]. Further, stigma associated with mental illness may lead to underreporting and undertreatment of PPD, perhaps particularly concerning more traditional or rural communities.

The present review is guided by the biopsychosocial model of health, which conceptualizes postpartum depression as the outcome of dynamic interactions between biological, psychological, and social determinants. From a biological perspective, rapid hormonal fluctuations, genetic susceptibility, and obstetric complications may increase maternal vulnerability. Psychological influences include cognitive appraisal, previous psychiatric history, and the availability of effective coping strategies, all of which can shape emotional resilience in the postpartum period. Social determinants, such as the level of family and partner support, prevailing cultural expectations, and socioeconomic status, may either buffer or intensify the impact of stressors. The categorization of risk factors into demographic, obstetric, psychosocial, and health-related domains reflect this framework and allows alignment with existing international evidence. In the Saudi context, where extended family living arrangements, marital relationship dynamics, and stigma associated with mental illness are important societal features, the biopsychosocial model offers a comprehensive lens for interpreting both the observed prevalence estimates and the identified risk factors.

The absence of a comprehensive synthesis of evidence on PPD in Saudi Arabia presents a significant gap in both research and practice. While several primary studies have explored the topic, their findings vary widely in terms of prevalence, risk factors, methodology, and clinical relevance. To the best of our knowledge, no systematic review and meta-analysis has yet been conducted to estimate the national prevalence and identify the risk factors of postpartum depression among Saudi women. Therefore, this study aims to estimate the prevalence and identify the risk factors associated with postpartum depression among Saudi women in Saudi Arabia.

## 2. Materials and Methods

The present systematic review and meta-analysis were conducted according to the guidelines presented in the Preferred Reporting Items for Systematic Review and Meta-analysis (PRISMA) 2020 statement [19]. This systematic review was retrospectively registered with the International Platform of Registered Systematic Review and Meta-analysis Protocols (INPLASY). The registration number is INPLASY202550064, and the DOI is 10.37766/inplasy2025.5.0064. Although the review process was initiated prior to formal registration, preliminary database searches and scoping exercises were undertaken to refine the research question and ensure sufficient availability of eligible studies before committing to full systematic review procedures. The protocol has been made publicly available through the INPLASY registry to enhance transparency and methodological rigor.

### 2.1. Information Sources

A search for topic-relevant literature was conducted on ProQuest, Web of Science, PubMed, and EBSCOHost, for articles published from inception to 31 March 2025. The following keywords were used together with Boolean operators (OR and AND) to generate search strings used in each database: “mothers”, “females”, “women”, “postpartum”, “puerperium”, “post-birth”, “postnatal”, “post birth”, “depressive”, “depression”, “PPD”, “depressed”, “prevalence”, “spread”, “risk factors”, “factor”, “risk”, “screening”, “Saudi Arabia”, and “Saudi”. The identified articles were subjected to a study selection process, as per the PRISMA guidelines, and as specified by predefined inclusion and exclusion criteria.

### 2.2. Eligibility Criteria

For a study to be eligible for inclusion in this review, it had to fulfil the following criteria: (i) include postpartum women residing in Saudi Arabia, primarily within the reproductive age group (18–49 years), regardless of age, parity, or region; the restriction to Saudi nationals reflects the inclusion criteria of the primary studies identified in the search, all of which recruited only Saudi women; (ii) include studies assessing risk factors potentially associated with postpartum depression; (iii) include studies reporting on the prevalence of postpartum depression and/or identifying risk factors associated with PPD using validated screening tools (e.g., Edinburgh Postnatal Depression Scale, Beck Depression Inventory, DSM criteria); (iv) allow for subgroup meta-analysis based on different EPDS cutoff points as a primary outcome; (v) be written in the English language.

Studies that focused exclusively on general depression without specifying or isolating PPD as a distinct condition were excluded. Non-original studies (e.g., reviews, editorials, meta-analyses, commentaries, other research papers, conference abstracts, or letters to the editor) were excluded.

Three independent reviewers (SZ, HA, and MF) screened the titles and abstracts of all identified studies. Full-text articles were obtained for those that met the inclusion criteria or where eligibility was unclear. Any disagreements were resolved by consensus or, if necessary, by consulting a fourth reviewer (MZ).

### 2.3. Data Extraction and Quality Appraisal

The extracted data included author and year of publication, study design, region, sample size, age (Mean ± SD), measurement tool, time of PPD assessment, prevalence of PPD (%), and cutoff score. The methodological quality of the studies was evaluated using the Joanna Briggs Institute (JBI) critical appraisal tools [20]. All studies were assessed independently by two reviewers (MF and HA) to be consistent and reduce bias. The JBI Critical Appraisal Checklist for Cohort Studies was used for cohort studies, which addresses important aspects of group comparability, measurement of outcomes, follow-up, and confounding. For cross-sectional studies, the JBI Checklist for Analytical Cross-Sectional Studies was utilized, addressing criteria including sampling, reliability of exposure and outcome measurement, and statistical analysis. Discrepancies among reviewers were dealt with by discussion or, if necessary, by consulting a third reviewer (SZ).

### 2.4. Statistical Analysis

Statistical analyses were conducted using R software (version 4.4.2) and Review Manager version 5.4. Subgroup analyses were performed to investigate the effects of assessment time points and the cutoff scores used for the Edinburgh Postnatal Depression Scale (EPDS) on the prevalence of postpartum depression (PPD). Studies were grouped based on comparable assessment intervals, specifically (i) 0–3 months and (ii) 4–12 months postpartum. Subgroup analyses were also conducted according to EPDS cutoff scores of 10, 12, 13, and 14 to compare prevalence estimates across varying symptom severity thresholds. To explore regional differences, further subgroup analyses were conducted based on the geographic location of the studies (central, western, eastern, and southern regions). For the analysis of risk factors associated with PPD, a random-effects model was also applied to pool the odds ratios (ORs) reported by the included studies, based on statistically significant associations. Heterogeneity was assessed using the I^2^ statistic, with values greater than 50% considered indicative of substantial heterogeneity. Funnel plots were used to visually assess potential publication bias. A *p*-value of <0.05 was considered statistically significant.

## 3. Results

### 3.1. Study Selection

**Figure 1:**
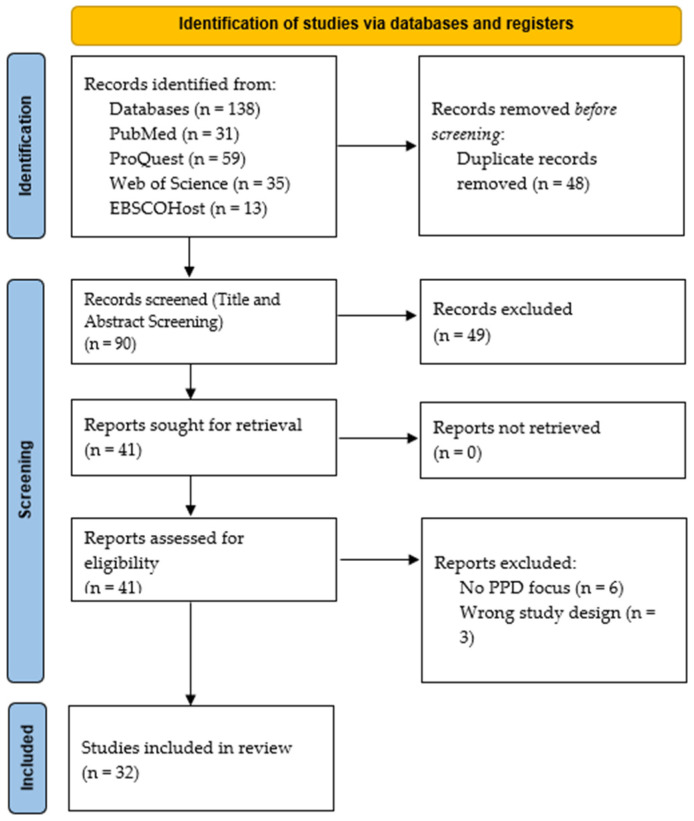
PRISMA flowchart showing search strategy and study selection.

The initial database search identified 138 articles: 31 from PubMed, 59 from ProQuest, 35 from Web of Science, and 13 from EBSCOHost. Ninety non-duplicates were assessed in the title and abstract screening, but 49 were excluded for non-eligibility. Forty-one articles were evaluated in the full-text review, and nine were excluded for various reasons, as shown below. Thirty-two studies were included in this review [Figure 1].

### 3.2. Results of Quality Assessment

We assessed the quality of the included studies using the Joanna Briggs Institute (JBI) tool, a widely recognized and validated instrument for evaluating the quality of research studies. For cohort studies, all three studies were assessed using the JBI tool [17,21,22], and each was found to have a low risk of bias [Supplementary Table S1]. Regarding the cross-sectional studies, the JBI tool for cross-sectional studies was used, and again, all studies received a good overall rating, indicating high quality [Supplementary Table S2].

### 3.3. Study Characteristics

This systematic review included a total of 32 studies (Table 1), comprising three cohort studies and 29 cross-sectional studies, with a combined population of 10,975 females. The sample size ranged from a minimum of 91 to a maximum of 1409. The average age of the participants was 30.38 ± 6.22 years. The studies were conducted across various regions of Saudi Arabia, including the Western region (Taif, Jeddah, Al-Madinah, and Makkah), the Central region (Riyadh, Qassim, and Al Kharj), the Southern region (Jizan, Najran, and Abha), and the Eastern region (Khobar and Dammam). The majority of the studies used EPDS (Edinburgh Postnatal Depression Scale) followed by BDI (Beck Depression Inventory) as measurement tools for PPD.

### 3.4. The Prevalence of PPD in Saudi Arabia

**Figure 2:**
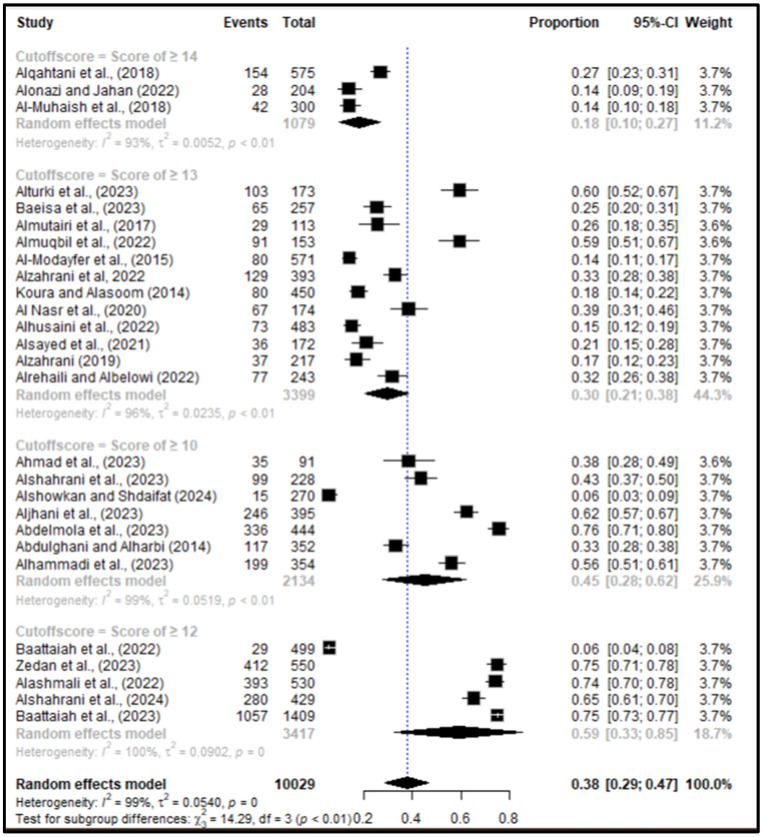
Subgroup analysis of PPD prevalence by EPDS cutoff scores. Studies have reported postpartum depression with a cutoff score of ≥10 [17,22,25,27,35,37,44]; ≥12 [30,42,43,45,47]; ≥13 [16,18,23,24,28,31,33,36,38,40,41,46]; and ≥14 [21,39,49].

Subgroup analysis was conducted based on different EPDS cutoff scores. When using an EPDS score ≥ 14, the pooled prevalence was 18% (95% CI: 10–27%, N = 1079). For studies using a cutoff of ≥13, the prevalence was 30% (95% CI: 21–38%, N = 3399). A cutoff of ≥12 resulted in a higher prevalence of 59% (95% CI: 33–85%, N = 3417), while a cutoff of ≥10 yielded a prevalence of 45% (95% CI: 28–62%, N = 2134). The overall test for differences among these subgroup cutoff scores was statistically significant (*p* < 0.01), indicating that the prevalence of postpartum depression varied significantly based on the EPDS threshold used [Figure 2].

**Figure 3:**
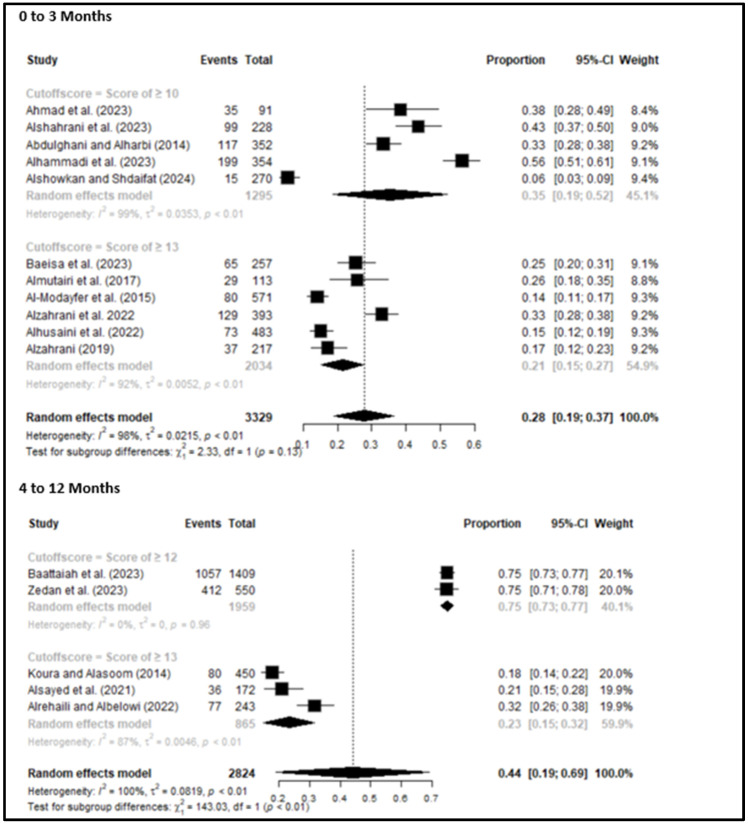
Subgroup analysis for prevalence of PPD at different assessment time points. In the 0–3 months: studies with cutoff ≥10 [17,22,25,27,44]; studies with cutoff ≥13 [16,24,28,33,38,41]. In the 4–12 months: studies with cutoff ≥12 [30,42]; studies with cutoff ≥13 [36,40,46]. To assess publication bias, funnel plots were examined. A slight asymmetry was observed, suggesting the possibility of publication bias [Supplementary Figure S1].

Subgroup analysis by time point showed notable differences in the prevalence of PPD depending on the assessment period. For women assessed within 0–3 months postpartum, five studies using an EPDS cutoff of ≥10 reported a pooled prevalence of 35% (95% CI: 19–52%, *p* < 0.01), while six studies using a cutoff of ≥13 showed a lower pooled prevalence of 21% (95% CI: 15–27%, *p* < 0.01). Among studies assessing PPD between 4–12 months postpartum, two studies using a cutoff of ≥12 reported a considerably higher pooled prevalence of 75% (95% CI: 73–77%, *p* = 0.96), whereas three studies with a cutoff of ≥13 found a prevalence of 23% (95% CI: 15–32%, *p* < 0.01) [Figure 3].

### 3.5. Regional Variations Analysis in PPD Prevalence

**Figure 4:**
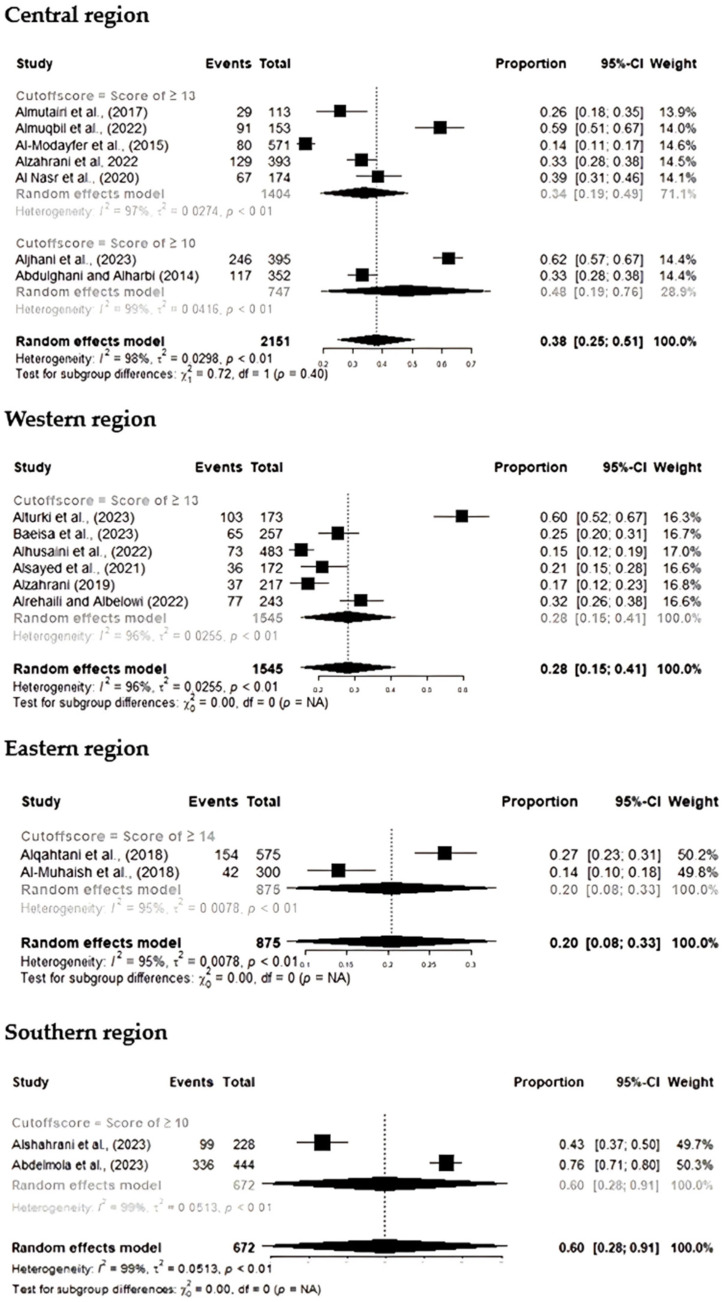
Subgroup analysis of regional variation prevalence by EPDS cutoff scores. Central region: studies with cutoff ≥13 [16,18,28,31,33]; studies with cutoff ≥10 [17,35]. Western region: studies with cutoff ≥13 [23,24,38,40,41,46]. Eastern region: studies with cutoff ≥14 [21,49]. Southern region: studies [25,37].

Subgroup analysis was performed to assess PPD prevalence across regions based on different EPDS cutoff scores. In the Central region, studies using a cutoff of ≥13 reported a prevalence of 34% (95% CI: 19–49%), while those using a cutoff of ≥10 reported a higher prevalence of 48% (95% CI: 19–76%). For the Western region, a cutoff of ≥13 resulted in a pooled prevalence of 28% (95% CI: 15–41%). The Eastern region had a prevalence of 20% (95% CI: 8–33%) when using a cutoff of ≥14, while the Southern region exhibited the highest prevalence, 60% (95% CI: 28–91%), for a cutoff of ≥10. The overall test for differences among both regional prevalence estimates and subgroup cutoff scores was statistically significant (*p* < 0.01) [Figure 4], indicating that PPD prevalence varied significantly across EPDS scoring thresholds.

### 3.6. Risk Factors Associated with PPD

A total of 32 risk factors for PPD were identified, all of which were statistically significant and categorized into four main groups: demographic, obstetric, psychosocial, and medical/health-related factors [Table 2]. Among the most frequently reported risk factors were lack of support (12 studies) and mode of delivery (11 studies). Demographic factors such as younger and older maternal age, low family income, and lack of employment were commonly associated with PPD. Obstetric factors, including pregnancy complications, unplanned pregnancy, and problems with the child’s health, also contributed to increased risk. Additionally, psychosocial factors such as stress, marital conflict, previous psychiatric history, and sleep disturbances were frequently reported. Medical-related factors, particularly a family history of depression (seven studies) and diseases during pregnancy (four studies), were also identified as significant contributors.

**Figure 5:**
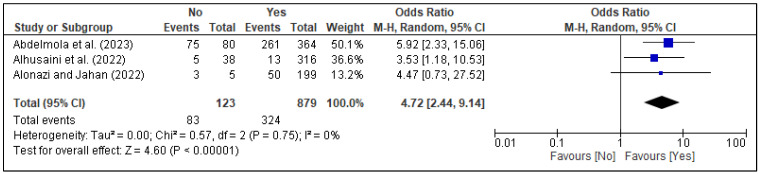
Forest plot for pooled association between family support and postpartum depression, based on data from studies [37,38,39].

**Figure 6:**
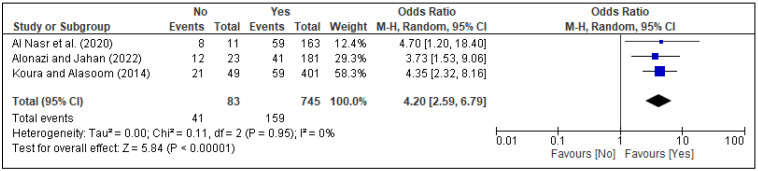
Forest plot for pooled association between spouse support and postpartum depression, based on data from studies [18,36,39].

**Figure 7:**
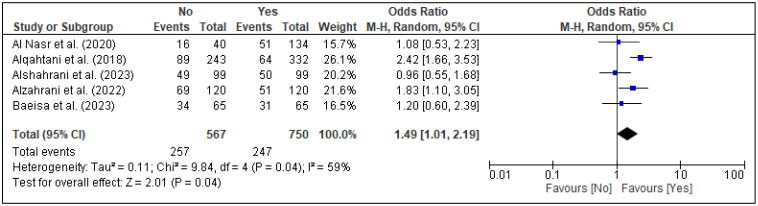
Forest plot for pooled association between planned pregnancy and postpartum depression, based on data from studies [18,21,24,25,33].

**Figure 8:**
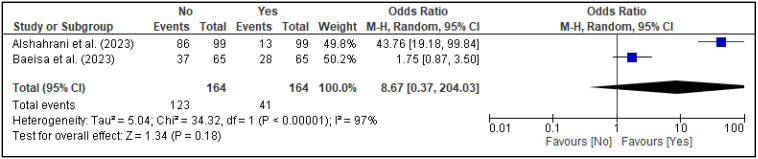
Forest plot for pooled association between complications during pregnancy and postpartum depression, based on data from studies [24,25].

**Figure 9:**
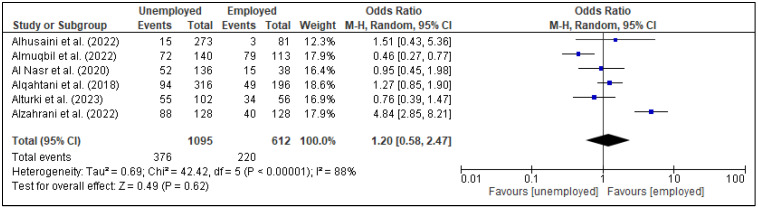
Forest plot for pooled association between employment status and postpartum depression, based on data from studies [18,21,23,31,33,38].

**Figure 10:**
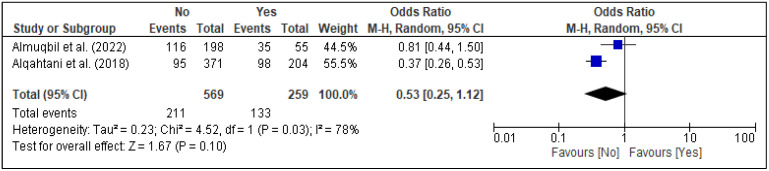
Forest plot for pooled association between history of miscarriage and postpartum depression, based on data from studies [21,31].

**Figure 11:**
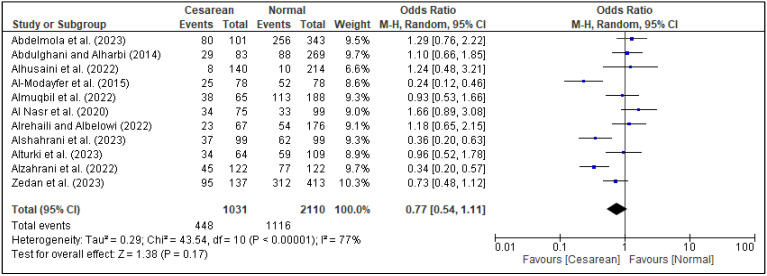
Forest plot for pooled association between mode of delivery and postpartum depression based on data from studies [16,17,18,23,25,31,33,37,38,42,46].

In the meta-analysis, three factors, i.e., family support [Figure 5], spouse support [Figure 6], and planned pregnancy [Figure 7], showed significant association with PPD, while the rest of the factors showed insignificant associations with PPD (Figure 8, Figure 9, Figure 10 and Figure 11). Family support was evaluated in three studies, and the pooled analysis revealed that women who lacked support from their families had significantly higher odds of developing PPD (OR = 4.72, 95% CI: [2.44–9.14] [Figure 5], *p* < 0.00001, I^2^ = 0%). Similarly, lack of spouse support was analyzed in three studies and was also associated with higher odds of PPD (OR = 4.20, 95% CI: [2.59–6.79] [Figure 6], *p* < 0.00001, I^2^ = 0%), while five studies assessed the impact of planned pregnancy on PPD, showing that women with unplanned pregnancies had significantly higher odds of experiencing PPD (OR = 1.49, 95% CI: [1.01–2.19] [Figure 7], *p* = 0.04, I^2^ = 59%).

## 4. Discussion

Postpartum depression is not only common but deeply variable depending on how—and where—it is measured. In this systematic review and meta-analysis, we found that the prevalence of postpartum depression among Saudi women varied widely depending on the EPDS cutoff score used. At a cutoff of ≥14, the pooled prevalence was 18%, while the prevalence increased to 30% with a cutoff of ≥13, 59% with ≥12, and 45% with ≥10. Subgroup analysis by assessment time points also revealed variation in prevalence: studies assessing PPD at 0–3 months postpartum reported lower prevalence than those at 4–12 months. These findings highlight how the choice of diagnostic threshold significantly influences reported prevalence rates. Regional subgroup analyses further revealed notable geographic disparities. The Southern region exhibited the highest prevalence (60%), followed by the Central (48%), Western (28%), and Eastern (20%) regions, with estimates again influenced by varying EPDS cutoffs. We identified 32 risk factors associated with PPD across the included studies. A meta-analysis revealed that lack of family support, spouse support, and unplanned pregnancy were significantly associated with increased odds of PPD, while other factors showed insignificant associations.

Compared with global estimates of 13% to 19% [5], the findings of this review suggest that postpartum depression among Saudi women may be substantially higher, depending on the assessment threshold used. Although no single overall prevalence was computed, stratified analysis by EPDS cutoff scores revealed a wide range of prevalence estimates—from 18% (EPDS ≥ 14) to 59% (EPDS ≥ 12)—underscoring the significant impact of methodological differences in screening and the potentially elevated burden of PPD in the Saudi population. In a very recent global systematic review by Shorey et al. [7], the overall estimated 17.7% reflected a disproportionately higher burden in Saudi women. Cultural, social, and health system-specific reasons in Saudi Arabia, such as the stigma of mental illness, restricted access to postpartum mental health care, and quality of regional support systems, may be reasons for the higher reported prevalence. Observed regional heterogeneity in PPD prevalence in Saudi Arabia is consistent with national studies and regional health reports. For instance, a previous study conducted in the Southern region also reported higher depressive symptomatology among postpartum women compared with other regions, potentially due to limited healthcare access, higher rates of unplanned pregnancies and low socioeconomic status [37]. In contrast, the Eastern region’s lower prevalence may reflect relatively better maternal health services and support systems in urbanized areas like Khobar and Dammam.

Regarding risk factors, this review reinforces commonly reported predictors of PPD globally, including lack of social support, stress, previous psychiatric history, and complications during pregnancy [50]. Notably, mode of delivery also emerged as a frequently cited risk factor among Saudi women, consistent with findings from international studies suggesting that cesarean sections are associated with a higher risk of postpartum mood disturbances [51,52]. The influence of demographic and psychosocial determinants is also consistent with global literature, yet the importance of cultural and familial dynamics in Saudi environments—e.g., conflict between spouses and extended family residence—deserves greater contextual investigation in future studies.

The psychosocial determinants of lack of support, conflict in the relationship, and prior history of psychiatric illness were the most reported, implying that social and emotional environments are crucial in determining maternal mental health. These results are especially pertinent in the Saudi population, where there are societal customs that may preclude disclosure of emotional suffering and mental illness, even in the postpartum condition, which may amplify solitude. Obstetric determinants, mode of delivery, complications in the course of labor, and unintended pregnancy were also frequently reported to coexist with the risk of PPD, indicating the role of both the physiological and emotional events of labor. Frequent mentions of caesarean delivery as a risk factor may be related to the psychological effects of unanticipated surgical intervention or concerns about recovery and caregiver capabilities; in Saudi Arabia, caesarean sections account for approximately 10% of all births, with rates reaching up to 20% in tertiary care centers [53]. Demographic factors such as younger age, low family income, and unemployment emphasize the social determinants of health and indicate that women with fewer resources may be particularly vulnerable. Finally, health-related conditions such as a family history of depression and medical illness during pregnancy suggest a biological susceptibility that intersects with social and environmental stressors.

### 4.1. Implications for Practice

These findings highlight the urgent need for routine screening of postpartum depression across all regions of Saudi Arabia, particularly in high-prevalence areas such as the Southern region. Incorporating the EPDS into postnatal care protocols, along with culturally sensitive mental health education and support systems, could facilitate early identification and intervention. Policymakers should prioritize maternal mental health by integrating psychological services into primary healthcare and designing community-based programs that address key risk factors, especially psychosocial stressors and limited social support.

### 4.2. Study Limitations

One of the main strengths of this review lies in its comprehensive inclusion of studies from multiple regions of Saudi Arabia, which allowed for a robust estimation of both national and regional prevalence rates of postpartum depression. The large sample size (10,975 women) across 32 studies strengthens the generalizability of the findings. Furthermore, the use of a validated and widely accepted tool—the EPDS—ensured methodological consistency. Subgroup analyses by EPDS cutoff scores provided insight into how different diagnostic thresholds influence prevalence estimates, contributing to a nuanced understanding of the burden of postpartum depression.

Despite these strengths, several limitations should be acknowledged. Due to heterogeneity in EPDS cutoff scores across studies, the meta-analysis focused primarily on subgroup-specific prevalence estimates rather than a single pooled national prevalence, as the latter would compromise the interpretability of the results. This approach provides a more clinically and methodologically coherent understanding of the postpartum depression prevalence in Saudi Arabia.

Additionally, most included studies were cross-sectional, which limits the ability to establish causality or temporal relationships between risk factors and PPD. While useful for estimating prevalence, cross-sectional designs are less robust than cohort studies, which provide stronger evidence due to their longitudinal nature.

Furthermore, visual assessment of funnel plots suggested possible publication bias, potentially skewing overall estimates. Another limitation is the underrepresentation of certain geographic regions, particularly the Northern region of Saudi Arabia, which restricts the ability to draw fully comprehensive national conclusions.

Regional variability may also influence prevalence estimates. The uneven regional distribution may introduce bias, as areas with fewer studies may be underrepresented in the subgroup-specific prevalence estimate.

Secondly, the inability to perform subgroup analyses by age, as most included studies reported PPD prevalence for the entire sample without stratifying by age groups.

Lastly, we could not meta-analyze all identified risk factors due to inconsistencies in how they were defined, measured, and reported across studies. Many lacked sufficient statistical detail or effect estimates, limiting comparability and restricting quantitative synthesis to only a subset of factors.

## 5. Conclusions

Postpartum depression remains a prevalent and pressing mental health concern among Saudi women, with reported rates ranging from 18% to 59% depending on the EPDS cutoff score used. The marked variation in prevalence by screening threshold and assessment timing underscores the importance of standardizing screening criteria in both research and clinical practice. Regional disparities were also evident, with the Southern region exhibiting the highest prevalence. These findings emphasize the influence of both methodological variation and geographic context on reported PPD rates. Subgroup analyses by assessment time further revealed that PPD prevalence tended to be higher at 4–12 months postpartum compared to earlier assessments, particularly when using a cutoff score of ≥12.

While 32 potential risk factors were identified, meta-analysis revealed that lack of family support, poor spouse support, and unplanned pregnancies were significantly associated with increased PPD risk. These findings highlight the central role of social and relational determinants in maternal mental health and indicate that preventive strategies should prioritize strengthening family and partner support networks, alongside providing counseling and family planning services.

By synthesizing data from 32 high-quality studies across all major regions of Saudi Arabia, this study provides the most comprehensive national evidence to date on PPD prevalence and risk factors. These findings not only confirm that PPD prevalence in Saudi Arabia is higher than global averages but also pinpoint modifiable factors that should be addressed first in prevention and intervention programs, such as culturally tailored mental health services and initiatives to strengthen family and partner support during the postpartum period. Future research should focus on generating higher-quality longitudinal data and formally quantifying the strength of associations for additional risk factors to better guide clinical and policy efforts.

## Figures and Tables

**Table 1:** The studies included in the systematic review and meta-analysis, along with their key characteristics.

Author and Year of Publication	Study Design	Region	Sample Size	Age (Mean ± SD) (Y)	Measurement Tool	Time of PPD Assessment (M)	Prevalence of PPD (%)	Cutoff Score
Al-Modayfer et al. [16]	Cross-sectional	Hospital, Central	571	NR	EPDS	0–3	14	Score of ≥13
Abdulghani and Alharbi, [17]	Cohort	Healthcare settings, Central	352	29.92 ± 5.7	EPDS	0–3	33.2	Score of ≥10
Al Nasr et al. [18]	Cross-sectional	Healthcare settings, Central	174	NR	EPDS	NR	38.5	Score of ≥13
Alqahtani et al. [21]	Cohort	King Fahd University Hospital, Eastern	575	NR	EPDS	NR	26.8	Score of ≥14
Alturki et al. [23]	Cross-sectional	Umm Al-Qura University, Western	173	NR	EPDS	NR	59.5	Score of ≥13
Ahmad et al. [22]	Cohort	King Abdulaziz University Hospital, Western	91	30.38 ± 5.09	EPDS	0–3	38.5	Score of ≥10
Baeisa et al. [24]	Cross-sectional	King Abdulaziz University Hospital, Western	257	31.6 ± 5.4	EPDS	0–3	25.3	Score of ≥13
Alshahrani et al. [25]	Cross-sectional	Abha City, Southern	228	30.9 ± 7.0	EPDS	0–3	43.4	Score of ≥10
Alshowkan et al. [26]	Cross-sectional	King Fahd University Hospital, Eastern	239	NR	NA	NR	NR	NR
Alshowkan and Shdaifat, [27]	Cross-sectional	Clinic of Gynaecology and Obstetrics, Eastern	270	NR	EPDS	6	5.6	Score of ≥10
Almutairi et al. [28]	Cross-sectional	Primary health care clinics, Central	113	NR	EPDS	0–3	25.7	Score of ≥13
Alharbi et al. [29]	Cross-sectional	Riyadh and Madinah, Central and Western	92	NR	HADS	0–3	NR	Score of ≥11
Baattaiah et al. [30]	Cross-sectional	King Abdulaziz University Hospital, Western	499	29.5	EPDS	NR	5.8	Score of ≥12
Almuqbil et al. [31]	Cross-sectional	Hospital, Central	153	31.45 ± 6.61	EPDS	NR	59.68	Score of ≥13
Aljaffer et al. [32]	Cross-sectional	Tertiary Care Hospital, Central	187	NR	EPDS	0–3	50.3	Score of ≥9
Alzahrani et al. [33]	Cross-sectional	Primary healthcare centres and military hospitals, Central	393	31.91 ± 6.45	EPDS	0–3	32.8	Score of ≥13
Al Rehaili et al. [34]	Cross-sectional	Primary healthcare centres, Western	252	31.3 ± 6.2	EPDS	0–3	NR	NR
Aljhani et al. [35]	Cross-sectional	Qassim, Central	395	NR	EPDS	4–12	62.3	Score of ≥10
Koura and Alasoom, [36]	Cross-sectional	Primary Health Care, Eastern	450	27.8 ± 5.4	EPDS	4–12	17.8	Score of ≥13
Abdelmola et al. [37]	Cross-sectional	Jizan Region, Southern	444	NR	EPDS	NR	75.7	Score of ≥10
Alhusaini et al. [38]	Cross-sectional	King Abdulaziz University Hospital, Western	483	31.58 ± 5.8	EPDS	0–3	15.1	Score of ≥13
Alonazi and Jahan, [39]	Cross-sectional	Primary Health Care Centres, Qassim, Central	204	NR	EPDS	NR	13.7	Score of ≥14
Alsayed et al. [40]	Cross-sectional	Primary healthcare centres, Western	172	29 ± 5	EPDS	4–12	20.9	Score of ≥13
Alzahrani, [41]	Cross-sectional	Western Region	217	33.2 ± 4.52	EPDS	0–3	17.1	Score of ≥13
Zedan et al. [42]	Cross-sectional	Whole Saudi Arabia	550	29 ± 5.4	EPDS	4–12	75	Score of ≥12
Alashmali et al. [43]	Cross-sectional	Whole Saudi Arabia	530	NR	EPDS	NR	74.11	Score of ≥12
Alhammadi et al. [44]	Cross-sectional	Whole Saudi Arabia	354	30.1 ± 6.78	EPDS	0–3	56.2	Score of ≥10
Alshahrani et al. [45]	Cross-sectional	Southern	420	NR	EPDS	0–3	66.7	Score of ≥12
Alrehaili and Albelowi, [46]	Cross-sectional	Academy of Family Medicine, Western	243	28.21 ± 11.54	EPDS	4–12	31.68	Score of ≥13
Baattaiah et al. [47],	Cross-sectional	Whole Saudi Arabia	1409	29 ± 5	EPDS	4–12	75	Score of ≥12
Elmagd and Albokhary, [48]	Cross-sectional	General hospitals, Central	185	28.7 ± 4.6	BDI	0–3	NR	NR
Al-Muhaish et al. [49]	Cross-sectional	Eastern	300	33.2 ± 9.2	EPDS	NR	14	Score of ≥14

Abbreviations: EPDS: Edinburgh Postnatal Depression Scale; BDI: Beck Depression Inventory; PPD: Postpartum depression; NR: Not reported; M: Months; Y: Years.

**Table 2:** Risk factors for PPD reported by the included studies.

Risk Factors	Variables	Definition	Number of Studies Reporting Risk for PPD
1. Demographic Factors	Younger age	Age < 25 years *	8
	Older age	Age ≥ 35 years *	6
	Socioeconomic status	Income, occupation, and education-based classification	1
	Type of family: nuclear and extended	Living in nuclear (parents + children) vs. extended (includes relatives) family	1
	Family income	Self-reported low vs. moderate/high income	5
	Level of education: Low	Primary/secondary education or less	4
	Level of education: High	University degree or above	3
	Lack of employment	Unemployed or not engaged in paid work	4
	Difficult life events	Stressful events prior to or during pregnancy (e.g., loss, financial stress)	3
	Occupation	Type of work or employment sector	3
2. Obstetric Factors	History of miscarriage	One or more previous miscarriages	1
	Thought that the pregnancy would negatively impact their life and work	Self-reported perception of burden or disruption	1
	Mode of delivery	Vaginal vs. cesarean delivery	11
	Pregnancy complications	Conditions like preeclampsia, gestational diabetes, etc.	6
	Multiple pregnancies	Twin or higher-order pregnancy	1
	Unplanned pregnancy	Pregnancy reported as unintended or mistimed	4
	Number of abortions	One or more previous abortions	2
	Parity	Number of previous births	4
	Delivery time	Daytime vs. nighttime delivery	1
	Attitude towards pregnancy	Positive vs. negative self-reported feelings	1
	Problems with the child’s health	Neonatal illness, complications, or disability	2
3. Psychosocial Factors	Lack of support	Inadequate social or family support during postpartum	12
	History of violence	Prior exposure to physical or emotional abuse	1
	Marital conflict	Ongoing disagreement or instability in relationship	2
	Stress	Self-reported high stress levels during or after pregnancy	6
	Previous psychiatric history	Any diagnosed mental health condition before pregnancy	6
	Sleep disturbance	Difficulty sleeping or poor sleep quality postpartum	3
	Anhedonia	Loss of interest or pleasure	1
	Mood swings	Reported emotional instability	1
4. Medical/Health-Related Factors	Family history of depression	Depression in first-degree relatives	7
	Disease during pregnancy	Physical illness during pregnancy	4
	Nutrition and Dietary factors	Reported poor diet, deficiencies, or eating habits	3

* Note: The definitions of “younger age” and “older age” were standardised for the purposes of synthesis. “Younger age” was defined as <25 years, “older age” as ≥35 years, and the reference age group as 25–34 years.

## Data Availability

The data that support the findings of this study are available within the article and its Supplementary Material.

## Supplementary Materials

The following supporting information can be downloaded at https://www.mdpi.com/article/10.3390/healthcare13162040/s1, Figure S1: Funnel plot showing Subgroup analysis of PPD prevalence by EPDS cutoff scores. Table S1: Quality results for cohort studies. Table S2: Quality results for cross-sectional studies.

## Abbreviations

The following abbreviations are used in this manuscript:

PPD	Postpartum Depression
EPDS	Edinburgh Postnatal Depression Scale
GCC	Gulf Cooperation Council (GCC)
BDI	Beck Depression Inventory
