# Supplementary material for: The Prevalence and Risk Factors of Postpartum Depression Among Saudi Arabian Women—A Systematic Review and Meta-Analysis
Source: Healthcare (Basel). 2025 Aug 18;13(16):2040. doi: 10.3390/healthcare13162040 (PMC12385902; doi:10.3390/healthcare13162040)
Supplement: Supplementary file 1 [file healthcare-13-02040-s001.zip › Manuscript Suplementary Tables.pdf]

Supplementary Table S1: Quality results for cohort studies

Study	Item 1	Item 2	Item 3	Item 4	Item 5	Item 6	Item 7	Item 8	Item 9	Item 10	Item 11	Overall
Alqahtani et al., (2018) [21]	NA	NA	Yes	Yes	Yes	Yes	Yes	No	No	Yes	Yes	Good
Ahmad et al., (2023) [23]	NA	NA	Yes	Yes	Yes	Yes	Yes	Yes	Yes	Yes	Yes	Good
Abdulghani and Alharbi (2014) [17]	NA	NA	Yes	Yes	Yes	Yes	Yes	Yes	Yes	Yes	Yes	Good

Item 1: Were the two groups similar and recruited from the same population? Item 2: Were the exposures measured similarly to assign people to both exposed and unexposed groups? Item 3: Was the exposure measured in a valid and reliable way? Item 4: Were confounding factors identified? Item 5: Were strategies to deal with confounding factors stated? Item 6: Were the groups/participants free of the outcome at the start of the study (or at the moment of exposure)? Item 7: Were the outcomes measured in a valid and reliable way? Item 8: Was the follow up time reported and sufficient to be long enough for outcomes to occur? Item 9: Was follow up complete, and if not, were the reasons to loss to follow up described and explored? Item 10: Were strategies to address incomplete follow up utilized? Item 11: Was appropriate statistical analysis used?

Supplementary Table S2: Quality results for cross-sectional studies

Study	Item 1	Item 2	Item 3	Item 4	Item 5	Item 6	Item 7	Item 8	Overall
Alturki et al. (2023) [22]	Ye	Yes	Yes	Yes	Yes	Yes	Yes	Yes	Good
Baeisa et al., (2023) [24]	Yes	Yes	Yes	Yes	Yes	Yes	Yes	Yes	Good
Alshahrani et al., (2023) [25]	Yes	Yes	Yes	Yes	Yes	Yes	Yes	Yes	Good
Alshowkan et al., (2023) [26]	Yes	Yes	Yes	Yes	No	No	Yes	Yes	Good
Alshowkan and Shdaifat (2024) [27]	Yes	Yes	Yes	Yes	Yes	Yes	Yes	Yes	Good
Almutairi et al., (2017) [28]	Yes	Yes	Yes	Yes	Yes	Yes	Yes	Yes	Good
Alharbi et al., (2024) [29]	Yes	Yes	Yes	Yes	Yes	Yes	Yes	Yes	Good

Baattaiah et al., (2022) [30]	Yes	Yes	Yes	Yes	Yes	Yes	Yes	Yes	Good
Almuqbil et al., (2022) [31]	Yes	Yes	Yes	Yes	No	No	Yes	Yes	Good
Al-Modayfer et al., (2015) [16]	Yes	Yes	Yes	Yes	No	No	Yes	Yes	Good
Aljaffer et al., (2023) [32]	Yes	Yes	Yes	Yes	No	No	Yes	Yes	Good
Alzahrani et al., (2022) [33]	Yes	Yes	Yes	Yes	Yes	Yes	Yes	Yes	Good
Al Rehaili et al., (2023) [34]	Yes	Yes	Yes	Yes	No	No	Yes	Yes	Good
Aljhani et al., (2023) [35]	Yes	Yes	Yes	Yes	Yes	Yes	Yes	Yes	Good
Koura and Alasoom (2014) [36]	Yes	Yes	Yes	Yes	No	No	Yes	Yes	Good
Abdelmola et al., (2023) [37]	Yes	Yes	Yes	Yes	No	No	Yes	Yes	Good
Al Nasr et al., (2020) [18]	Yes	Yes	Yes	Yes	Yes	Yes	Yes	Yes	Good
Alhusaini et al., (2022) [38]	Yes	Yes	Yes	Yes	No	No	Yes	Yes	Good
Alonazi and Jahan (2022) [39]	Yes	Yes	Yes	Yes	No	No	Yes	Yes	Good
Alsayed et al., (2021) [40]	Yes	Yes	Yes	Yes	Yes	Yes	Yes	Yes	Good
Alzahrani (2019) [41]	Yes	Yes	Yes	Yes	No	No	Yes	Yes	Good
Zedan et al., (2023) [42]	Yes	Yes	Yes	Yes	No	No	Yes	Yes	Good
Alashmali et al., (2022) [43]	Yes	Yes	Yes	Yes	No	No	Yes	Yes	Good

Alhammadi et al., (2023) [44]	Yes	Yes	Yes	Yes	No	No	Yes	Yes	Good
Alshahrani et al., (2024) [45]	Yes	Yes	Yes	Yes	No	No	Yes	Yes	Good
Alrehaili and Albelowi, (2022) [46]	Yes	Yes	Yes	Yes	No	No	Yes	Yes	Good
Baattaiah et al., (2023) [47]	Yes	Yes	Yes	Yes	Yes	Yes	Yes	Yes	Good
Elmagd and Albokhary (2021) [48]	Yes	Yes	Yes	Yes	No	No	Yes	Yes	Good
Al-Muhaish et al., (2018) [49]	Yes	Yes	Yes	Yes	No	No	Yes	Yes	Good

Item 1: Were the criteria for inclusion in the sample clearly defined? Item 2: Were the study subjects and the setting described in detail? Item 3: Was the exposure measured in a valid and reliable way? Item 4: Were objective, standard criteria used for measurement of the condition? Item 5: Were confounding factors identified? Item 6: Were strategies to deal with confounding factors stated? Item 7: Were the outcomes measured in a valid and reliable way? Item 8: Was appropriate statistical analysis used?
