# Supplementary material for: The Prevalence and Risk Factors of Postpartum Depression Among Saudi Arabian Women—A Systematic Review and Meta-Analysis
Source: Healthcare (Basel). 2025 Aug 18;13(16):2040. doi: 10.3390/healthcare13162040 (PMC12385902; doi:10.3390/healthcare13162040)
Supplement: Supplementary file 1 [file healthcare-13-02040-s001.zip › Manuscript Supplementary Figure S1.pdf]

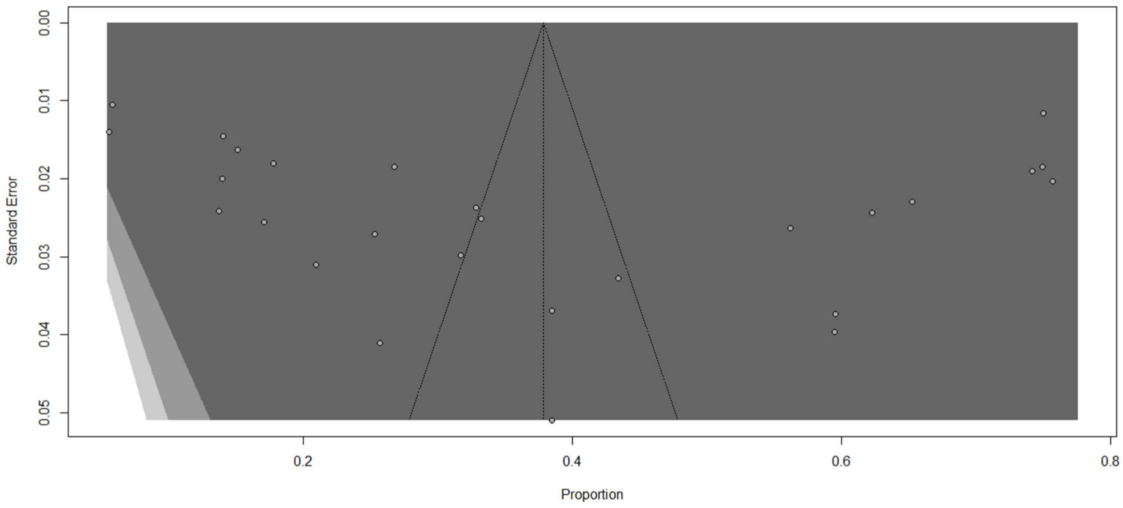


Supplementary Figure S1: Funnel plot showing Subgroup analysis of PPD prevalence by EPDS cutoff scores
